# Editorial: From reconstituting minimal cell-cell signaling systems to bioinspired synthetic communication networks

**DOI:** 10.3389/fmolb.2022.979163

**Published:** 2022-08-04

**Authors:** Kristina A. Ganzinger, Claudia Bonfio, Allen P. Liu

**Affiliations:** ^1^ AMOLF, Amsterdam, Netherlands; ^2^ CNRS, UMR 7006, Institut de Science et d’Ingénierie Supramoléculaires (ISIS), Université de Strasbourg, Strasbourg, France; ^3^ Department of Mechanical Engineering, University of Michigan, Ann Arbor, MI, United States; ^4^ Department of Biomedical Engineering, University of Michigan, Ann Arbor, MI, United States; ^5^ Department of Biophysics, University of Michigan, Ann Arbor, MI, United States; ^6^ Cellular and Molecular Biology Program, University of Michigan, Ann Arbor, MI, United States

**Keywords:** synthetic biology, artificial cell, signalling, minimal signalling systems, cell-cell communication

The field of constructing artificial/synthetic cells from non-living materials to emulate some aspect of natural cell functions has come a long way. The review by Wang et al. describes the next phase of artificial cell research of integrating different functional modules. They divided the functional requirements of an artificial cell into four essential modules: metabolism, energy supplement, proliferation, and communication. Substance metabolism promotes dynamic communication among substrates, allowing macromolecules to be synthesized. To maintain sustainable metabolism, energy in the form of ATP needs to be supplied or generated. With energy and metabolism in place, autonomous proliferation of artificial cells becomes feasible. There have been several recent advances in this module where growth of artificial cells with membrane addition and artificial cell “division” based on protein self-assembly or membrane mechanics have been demonstrated. The final module of communication will endow artificial cells with sense and response capabilities, both in controlling their own biochemistry and for communicating between artificial cells.

The mini-review by Smith et al. extends the concept of controlling synthetic cell communication further. The motivation to focus on communication is the potential to interface synthetic cells with living cells or to assemble interconnected synthetic cells to perform more complex tasks as a collective. Synthetic cells that respond to either chemical inputs or physical inputs have been demonstrated. Regulated expression of channel proteins allows synthetic cells to communicate using small molecule inducers (i.e., genetically encoded) or pH-based chemical conformational changes (i.e., non-genetic control). Physical stimuli such as light, temperature, acoustics, or magnetic field can enable spatiotemporal control of synthetic cell communication. In particular, optogenetic activation of gene expression is a promising approach, though there is a need to develop light-activated systems that respond to longer wavelengths of light. Given the wide range of genetic circuits designed to enable stimuli-responsive behaviors in living cells, translating them to synthetic cells will be a major direction and could result in transformative advances in the application of synthetic cells in biomedical contexts.

The development and engineering of artificial cell networks capable of exchanging chemical signals have the potential to lead to significant scientific breakthroughs and biotechnological applications in regenerative medicine and tissue engineering. In their mini-review, Grimes et al. collect and discuss efforts in this novel, trending area of synthetic biology, describing three categories of bioinspired communicating artificial cell networks. First, the authors report on the successful attempts at achieving communication between individual populations of protocells, explaining the strategies and methods employed for the sensing, processing and sending of chemical signals by synthetic cells. The authors later expand this concept by highlighting recent work on chemical communication between interrelated protocell networks. The last section of this minireview focuses on future trajectories and potential advances towards achieving chemical communication between synthetic and living cells.

To summarize the state-of-the-art in molecular communication between (responsive) artificial cells, Karoui et al. define the employed nomenclature, discuss the main design approaches and share future perspectives in the field of chemical artificial cell signaling. The initial focus is on the basic principles and concepts required to build an artificial cell capable of sending and sensing a chemical signal. A thorough description of the various reports on artificial cell signaling is organized according to the distance between the involved cells and the mode of communication. The authors then proceed to discuss examples of artificial cells capable of dynamic, responsive and adaptive communication, as well as recent advances in collective behaviors enabled by signaling.

In summary, the field has come a long way in recent years to make communities of “communicating” synthetic cells a reality. There are now many ways to implement chemical communication between synthetic cells beyond the exchange of DNA messenger molecules, for example combining stimuli-sensing pores with molecular transport of protein inhibitors or activators. Clearly, many challenges still lie ahead: even the best synthetic signaling systems lack the information processing capacity achieved by living cells or tissues. Pushing towards ever more complex systems, for example by combining many of the currently developed strategies, should lead to many exciting findings and applications in the years to come.

